# Network Pharmacology Analysis of Liquid-Cultured *Armillaria ostoyae* Mycelial Metabolites and Their Molecular Mechanism of Action against Gastric Cancer

**DOI:** 10.3390/molecules29071668

**Published:** 2024-04-08

**Authors:** Zhishuo Wang, Ruiqi Wang, Zhiguo Na, Shanshan Liang, Fan Wu, Hongyao Xie, Xue Zhang, Wei Xu, Xin Wang

**Affiliations:** 1 School of Food Engineering, Harbin University of Commerce, Harbin 150028, China; yeah123123456@163.com (Z.W.); ruiqiwang2012@163.com (R.W.); nzgfood@163.com (Z.N.); 18804618842@163.com (S.L.); wfwyxy117118@163.com (F.W.); xhy2022001@163.com (H.X.); z12340605@163.com (X.Z.); 2 Key Laboratory for Food Science and Engineering, Harbin University of Commerce, Harbin 150028, China

**Keywords:** *Armillaria ostoyae*, liquid-cultured mycelium, ethanol extract, network pharmacology, molecular docking, gastric cancer

## Abstract

*Armillaria* sp. are traditional edible medicinal mushrooms with various health functions; however, the relationship between their composition and efficacy has not yet been determined. Here, the ethanol extract of liquid-cultured *Armillaria ostoyae* mycelia (AOME), a pure wild *Armillaria* sp. strain, was analyzed using UHPLC-QTOF/MS, network pharmacology, and molecular docking techniques. The obtained extract affects various metabolic pathways, such as JAK/STAT and PI3K/AKT. The extract also contains important compounds such as 4-(dimethylamino)-N-[7-(hydroxyamino)-7-oxoheptyl] benzamide, isoliquiritigenin, and 7-hydroxycoumarin. Moreover, the extract targets key proteins, including EGFR, SCR, and IL6, to suppress the progression of gastric cancer, thereby synergistically inhibiting cancer development. The molecular docking analyses indicated that the main compounds stably bind to the target proteins. The final cell culture experimental data showed that the ethanol extract inhibited MGC-803 gastric cancer cells. In summary, our research revealed the beneficial components of AOME for treating gastric cancer and its associated molecular pathways. However, further research is needed to confirm its effectiveness and safety in gastric cancer patients.

## 1. Introduction

Medicinal mushrooms are a class of natural fungal resources that are good sources of nutrients and functional active ingredients [1]. More than 2000 species of mushrooms have been identified for use as food or for medicinal purposes [2]. *Armillaria*, a widely distributed genus of edible and therapeutic mushrooms, is classified under Basidiomycetes, Agaricales, and Tricholomataceae, and can be found in Europe and Asia. This genus was originally widely distributed in Europe, Asia, North America, and many other regions, as well as tropical and temperate forest areas. In China, it is mainly produced in Heilongjiang, Jilin, Liaoning, Hebei, and other places [3]. The *Armillaria* sp. is composed of two main parts: the mycelium and the fruiting body. The mycelium exists in two forms: hyphae and rhizomorphs. It is a well-known edible fungus and a root rot pathogen that harms forests [4]. In clinical settings, the fermented products of artificially cultured mycelium have shown multifaceted pharmacological activities in the nervous and cardiovascular systems [5]. More than 70 sesquiterpenoids have been reported from artificially fermented mycelium in *Armillaria* sp. [6], along with other compounds such as flavonoids, alkaloids, purines, and fatty acids. Modern analytical techniques have led to the discovery of additional compounds in artificially cultured mycelium, prompting further study of the relationship between active compounds and their functions by domestic and foreign scholars. In recent years, there have been several publications on the suppression of cancer cells using different compounds found in artificially fermented mycelium of the *Armillaria* sp. As an example, armillaridin is an aromatic ester compound that has been isolated from *Armillaria* sp. It has the ability to inhibit the differentiation and activation of macrophages by suppressing their phagocytic activity [7]. Armillaridin can hinder the proliferation of Huh7, HepG2, and HA22T hepatocellular carcinoma cells through the disruption of the mitochondrial transmembrane potential. Additionally, armillaridin can promote the death of HCC cells through autophagy, as stated in reference [8]. In addition, armillaridin has been found to inhibit the growth of human chronic granulocytic leukemia K562 cells and induce autophagy-associated cell death [9]. The polysaccharide structure of honeydew is primarily composed of D-glucose. Previous studies suggest that *Armillaria mellea* polysaccharides may have antitumor activity by inducing apoptosis and cell blockade in A549 cells. Additionally, the mitochondria-dependent pathway was demonstrated, showing that cytochrome c release induced the activation of caspase-3 and -9 signaling [10].

Due to the limited sources of *Armillaria* sp. in natural forests, this study used a pure parent strain for the artificial liquid culture of the mycelia. This process can be performed on a large scale for the industrial production of mycelia and to solve the problem of insufficient natural resources. At present, there has been no comprehensive analysis of the components contained in artificially cultured mycelia, and clarifying the relationship between mycelium composition and efficacy is an urgent research problem. In this respect, network pharmacology can be used to generate complex interaction networks, which are highly important for screening effective compounds, performing preclinical research, and elucidating the possible mechanisms of action of natural compounds.

In this study, the pure parent strain (identified as *Armillaria ostoyae*, *A. ostoyae*) was isolated from the wild *Armillaria* sp. substrates harvested from the Heilongjiang forest area in China and artificially inoculated in liquid media for mycelial culture. Using UHPLC-QTOF/MS combined with network pharmacology and molecular docking, functional compounds with anti-gastric-cancer effects were screened from secondary metabolites, and the multitargeted mechanism of the *Armillaria ostoyae* mycelium ethanol extract (AOME) in gastric cancer was explored. To complete the initial investigations of the inhibitory impact of the AOME on cells, MGC-803 gastric cancer cells were ultimately chosen as the experimental subjects. This research examines fresh functional food ingredients made from edible mushrooms that have anti-gastric-cancer properties.

## 2. Materials and Methods

### 2.1. Materials

The *A. ostoyae* pure parent strain was obtained from the fruiting body of the untamed *Armillaria* sp., collected from Sunwu County, Heilongjiang Province, China. The pure parent strain was identified by morphology and molecular biology in the Applied Microbiology Laboratory of Food College (Harbin University of Commercial, Harbin, China). PDA, potato, sucrose, glucose, agar, yeast extract, peptone, MgSO_4_ꞏ7H_2_O, and KH_2_PO_4_ were obtained from Haibo Biotechnology Co., Ltd. (Hi-Tech Industrial Park, Qingdao, China). UPLC-grade reagents were obtained from Merck (Darmstadt, Germany). 

Wuhan HYcell Biological Co., Ltd. (Wuhan, China) provided the MGC-803 cell line.

### 2.2. Liquid Culture of A. ostoyae Mycelia

Modifications were made to the classical PDA medium used for cultivating mushrooms based on the results of previous laboratory studies. The optimal medium and culture process conditions were determined for cultivating liquid mycelium of *A. ostoyae*. These conditions resulted in a higher number of dry basidiomycetes, up to 1.36 g/100 mL. To create the media, 10.0 g of glucose, 5.0 g of peptone, 0.3 g of KH_2_PO_4_, 1.0 g of MgSO_4_ꞏ7H_2_O, and 1000 milliliters of distilled water were mixed together. The liquid was divided into 100 mL portions, and each portion was placed in a triangular bottle with a capacity of 250 mL. The sterilization was performed at 121 °C and 1.03 atm for 30 min. *A. ostoyae* (1 cm in diameter) was selected from the culture medium with a punch and inoculated in liquid medium. The culture bottles were placed in a shaker with a rotation speed of 160 r/min, at a temperature of 26 °C, and oscillated for 1–24 days.

### 2.3. Scanning Electron Microscopy (SEM) Observations of Mycelial Morphology

The mycelia of *A. ostoyae* were fixed in pH 6.8 glutaraldehyde fixative for 2 h in a refrigerator at 4 °C and subsequently rinsed twice for 10 min with a pH 6.8 phosphate buffer. After rinsing, the samples were dehydrated once with 50%, 70%, and 90% ethanol and subsequently dehydrated 3 times for 10 min with 100% ethanol. Afterward, the samples were immersed in a solution containing 100% ethanol and tert-butanol (1:1) for a duration of 1/10 min and then soaked in pure tert-butanol two times for 15 min each time. Subsequently, the specimens were placed at −20 °C for 30 min and subjected to freeze-drying. Finally, the samples were glued and coated to obtain test samples.

### 2.4. Sample Preparation

Mycelia, which were prepared following the procedure outlined in Section 2.2, were isolated from the fermentation broth through vacuum filtration, collected, and washed with flowing water. Then, the samples were obtained by drying at 60 °C, crushing, and passing through a 40 mesh sieve. An ultrasonic cell crusher was applied to the sieved sample powder, and the sample was heated to reflux in a Soxhlet apparatus at 60 °C for 50 min with ethanol as the solvent. After filtration, the solution was concentrated in a rotary evaporator with a water bath at 60 °C and 120 r/min. The solution was concentrated to obtain an AOME solution with a concentration of 0.128 g/mL.

A precisely calculated specimen weighing 60 milligrams was transferred into a 1.5 milliliter Eppendorf tube. Then, two tiny steel balls were added to the tube. A volume of eighty microliters of methanol-dissolved L-2-chlorophenylalanine (0.3 mg/mL), the internal standard, was introduced to each sample. Furthermore, 600 µL of a solution containing methanol and water in a ratio of 7 parts methanol to 3 parts water (volume/volume) was included. Subsequently, the samples were stored at −40 °C for 2 min. After subjecting the sample to a 2 min grinding session at a rate of 60 Hz, the complete specimen was extracted by means of ultrasonication for a duration of 30 min while placed in a bath of ice water. Afterward, the samples were stored overnight at −40 °C. Before pouring, the specimens were spun at a temperature of 4 °C (13,000 revolutions per minute) for a duration of 10 min. Then, using crystal syringes, 150 μL of the liquid above the sediment in each tube was collected, passed through 0.22 μm microfilters, and finally transferred to LC vials. The vials were kept at −80 °C for preparation.

### 2.5. UHPLC–QTOF/MS-Based Analysis of Secondary Metabolites in the A. ostoyae Mycelia

For analysis of the secondary compounds in the *A. ostoyae* mycelia, the Agilent 1290 Infinity UHPLC system (Agilent Technologies, Santa Clara, CA, USA) and Waters Acquity UPLC HSS T3 C18 column (2.1 mm × 100 mm, 2.1 μm; HSS T3; Waters, Milford, MA, USA) were used with a column temperature of 40 °C. A total of 2 μL was injected into the column at a 0.4 mL/min flow rate. For elution, the mobile phase was composed of aqueous 0.1% formic acid (component A) and acetonitrile containing 0.1% formic acid (component B). The improved gradient elution procedure was as follows: 0.0–11.1 min, 95% to 10% A; 11.0–12.0 min, 10% A; 12.0–12.1 min, 10% to 95% A; and 12.1–14.0 min, 95% A.

For analysis of the mass spectrometry, the Agilent Q-TOF/MS-6545 system (Agilent Technologies, USA) with an electrospray ionization (ESI) source was used. Both positive and negative ions were analyzed. Using the full-scan mode, the mass spectrometer was operated in the 100–1500 *m/z* range. The ideal parameters were established as follows: gas flow rate of 8 arb, sheath flow rate of 11 arb, gas temperature at 325 °C, sheath temperature at 325 °C, S-lens RF level at 40%, and spray voltage set at 250 V for (+)-ESI and 1.5 kV for (−)-ESI.

### 2.6. Network Pharmacology Analysis

Using the chemical component data obtained from the UHPLC-QTOF/MS analysis, combined with data obtained from the literature review and search results, a liquid *A. ostoyae* mycelium chemical component database was obtained. SwissADME [11] was used to screen the active ingredients.

Swisstarget prediction [12] was used to obtain the corresponding targets for the active ingredients in AOME. To detect genes linked to gastric cancer and forecast associated targets, we used various databases, including GeneCards [13], DrugBank [14], DisGeNET [15], PharmGKB [16], OMIM [17], NCBI, and TTD [18]. The keyword utilized was ‘gastric cancer’.

To filter the nodes, the network topology features of the nodes in the interaction network were examined by creating protein–protein interaction (PPI) networks with the help of BisoGenet [19] and CytoNCA plugins in Cytoscape 3.7.2. Five important topological parameters were utilized: betweenness [20], closeness [20], degree [21], a method based on local average connectivity [22], and network centrality [23]. For the Gene Ontology (GO) and Kyoto Encyclopedia of Genes and Genomes (KEGG) pathway analyses, we utilized the Metascape database [24]. GO analysis was conducted to identify the biological processes (BPs), cellular components (CCs) [25], and molecular functions (MFs). Ultimately, Cytoscape 3.7.2 software was used to establish a network that connected components, targets, and pathways.

### 2.7. Molecular Docking

We performed molecular docking to improve the accuracy and validate the binding between the key functional compounds in AOME and key targets [26]. Ligands were chosen from the AOME active ingredient target network, which consisted of the top 10 compounds. Furthermore, the top 3 targets were selected from both the PPI network that connected the drugs and diseases and the network of the main pathway targets. Schrödinger software (*Schrödinger* Release 2021-3) was used for ligand and protein file processing and to identify active sites and perform molecular docking. Compound–protein complexes with lower scores and free binding energies were considered to have greater binding stability according to the most accurate XP docking method. MM-GBSA was also used to calculate the active site.

### 2.8. Cytotoxicity Analysis

#### 2.8.1. Cell Culture and Treatment

Human-derived MGC-803 cells were cultivated in an RPMI-1640 medium (HyClone, Logan, UT, USA), supplemented with 10% fetal calf serum (Hangzhou Tianhang Biotechnology Co., Ltd., Hangzhou, Zhejiang, China) and 1% penicillin/streptomycin (HyClone, Logan, UT, USA) at 37 °C with 5% CO_2_. The experimental samples and the positive control 5-fluorouracil (5-FU) were dissolved in RPMI-1640 medium and filtered through a 0.22-micron filter to prepare the sample solutions. 

The experimental groups were as follows: (1) the blank control group: no sample was added; (2) the combined drug treatment groups: low (75 μg/mL AOME + 50 μg/mL 5-FU); medium (150 μg/mL AOME + 50 μg/mL 5-FU); and high extract concentrations (300 μg/mL AOME + 50 μg/mL 5-FU); and (3) the positive control group: 50 μg/mL 5-FU.

#### 2.8.2. Cell Viability Assay

To determine the MGC-803 cell viability, CCK-8 assays were conducted. For a duration of 1 to 4 h, the cells were exposed to AOME at concentrations ranging from 75 to 300 μg/mL. The calculation of the cell survival rate was as follows: cell survival rate (%) = (1−$\overset{—}{\mathrm{OD}}$_experimental group_/$\overset{—}{\mathrm{OD}}$_control group_) × 100%. The optical density (OD) values measured in each group allowed for the calculation of the two-drug interaction coefficient. The calculation of the CDI was as follows: CDI = AB/(A × B). In this case, AB denotes the ratio of OD in the combined drug group compared to that of the blank control group, A represents the ratio of OD of the sample group when used alone compared to that of the blank control group, and B represents the ratio of OD of the positive control group when used alone compared to that of the blank control group.

#### 2.8.3. Cell Adhesion Analysis

Following a 4 h period of drug administration, the adhesion of human MGC-803 cells was assessed using CCK-8 assays. The cell adhesion inhibition rate was as follows: Cell adhesion inhibition rate (%) = (1−$\overset{—}{\mathrm{OD}}$_experimental group_/$\overset{—}{\mathrm{OD}}$_blank control group_) × 100%.

#### 2.8.4. Wound Healing Analysis

To analyze wound healing, wounds were created in human MGC-803 cell cultures in 6-well plates by utilizing pipette tips when they reached approximately 80% confluence. Following gentle washing with phosphate-buffered saline (PBS; Aspen, Wuhan, Hubei, China), the cells were treated for 24 h. Subsequently, the wounds were directly examined using an inverted microscope. The cell mobility percentage was calculated by subtracting the initial scratch area from the scratch area after 24 h, dividing the result by the initial scratch area, and multiplying by 100%.

#### 2.8.5. Transwell Migration Analysis

As per the experimental grouping, the upper chamber of the Transwell plate contained 200 μL of MGC-803 cell suspension and 1 mL of RPMI-1640 medium containing different concentrations of the sample. Afterward, 500 μL of pretreated liquid containing 20% FBS was added to the lower compartment, which was subsequently incubated for 48 h at 37 °C. Afterward, the cells were washed and stained with crystal violet. The cells were counted under an inverted microscope to determine the extent of cell invasion inhibition. The inhibition percentage was calculated by subtracting the number of invading cells in the experimental group from the number of invading cells in the control group and dividing this value by the number of invading cells in the control group followed by multiplying the result by 100%.

#### 2.8.6. Immunoblotting

After treatment with the ether extract of *A. ostoyae* mycelia, human MGC-803 cells were suspended in lysis buffer (Aspen, Wuhan, Hubei, China) supplemented with protease inhibitors (Aspen, Wuhan, Hubei, China) for protein extraction. Following the removal and quantification of the debris, the cell lysates, each containing 40 μg of protein, were separated via 12% sodium dodecyl sulfate–polyacrylamide gel electrophoresis. Immunoblotting was performed using the following proteins: caspase-3 (Affbiotech, Liyang, Jiangsu, China), Bcl-2 (Abcam, Cambridge, UK), and GAPDH (loading control; Abcam, Cambridge, UK).

### 2.9. Data and Statistical Analyses

The original mass spectrometry data were converted into mzML format using Pro-teoWizard, and the peaks were extracted, aligned, and corrected using the XCMS program. The peak areas were corrected by the SVR method, and the peaks with a missing rate of more than 50% in each group were filtered. The screened peaks were corrected, and metabolite identification was performed by searching a self-built laboratory database and integrating the public database with the metDNA method. Finally, statistical analysis was carried out with the R program. The statistical analysis involved univariate and multivariate statistical analyses. Univariate statistical analysis included Student’s *t* test and multiple difference analysis, and multivariate statistical analysis included principal component analysis (PCA) and orthogonal partial least squares discriminant analysis (OPLS-DA). The Kyoto Encyclopedia of Genes and Genomes (KEGG) database (*URL*: http://www.genome.jp/kegg/pathway.html; accessed on 1 January 2023) was used to enrich and analyze the KEGG metabolic pathways. All the statistical analyses were performed using SPSS v.22.0 and Origin v.8.0 software. Based on normality and homogeneity of variance testing, one-way ANOVA was used for data analysis, and a *p* value < 0.05 was considered to indicate statistical significance.

## 3. Results

### 3.1. Growth Pattern of Pure Parent Strain in Solid Culture

**Figure 1:**
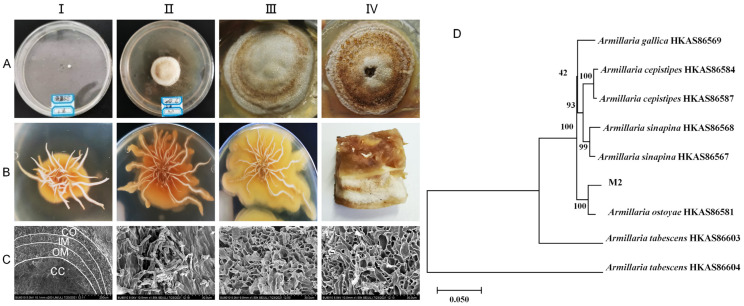
Growth status of pure parent strain in solid culture. (**A**)-(**I**–**IV**) show the colony morphology at days 3, 12, 24, and 42, respectively. (**B**)-(**I**–**III**) show the rhizomorph morphology at days 12, 24, and 42, respectively. (**B**)-(**IV**) is the longitudinal section of the colony at day 42. (**C**)-(**I**–**IV**) show the morphology of the transverse section of the whole rhizomorph, the cortex (CO), the outer medulla (OM), and the inner medulla (IM) portions, respectively, under scanning electron microscopy. CC represents the central cavity. (**D**): Phylogenetic tree constructed by three-sequence merger of pure parent strain (M2).

The mycelia of the pure parent strain were inoculated on PDA solid medium and cultured at 26 °C under dark conditions for 42 d to study the state of colony size change, color change, and the degree of bulging and hardness during this period. The growth patterns of the colony and rhizomorph are shown in Figure 1A,B, respectively. The observations on the solid plate colonies and the mycelia of the pure parent strain revealed that they sprouted on the third day. By the twelfth day, the surface of the colony appeared light honey-colored, with an elevated center and rhizomorph sprouting on the bottom surface. White droplets and a honey-colored pigment circle appeared on the twenty-fourth day, and by the forty-second day, the surface of the colony and droplets had almost entirely changed to a honey color. Upon further observation of the solid culture state of the inoculated young rhizomorph tip, it was found that after 12, 24, and 42 days of culture, the rhizomorph’s color remained unchanged. However, the extension state changed from straight to curved, the tip bifurcation changed from biaxial to uniaxial, and the colony’s surface changed from pure white to a honey-colored hue close to the culture medium. Upon further observation of the rhizomorphs at 42 d through electron microscopy, the complete structure of the rhizomorphs was clearly visible (Figure 1C). The structure was divided into four parts: cortex (CO), outer medulla (OM), inner medulla (IM), and central cavity (CC), from outside to inside.

The pure parent strain was characterized using molecular biology. A phylogenetic tree was constructed by combining the sequences of ITS, β-tubulin, and EF1-α (Figure 1D). The pure parent strain and *A. ostoyae* HKAS86581 from Dunhua, Jilin, were good sister lines, with a support rate of 100%. All the organisms of the same species were good sister lines to each other, with a Bootstrap value of ≥93%. The final identification result was *A. ostoyae*. The sequencing results for *A. ostoyae* are shown in Appendix A.

### 3.2. Mycelial Growth Pattern in the Artificial Liquid Culture of A. ostoyae

**Figure 2:**
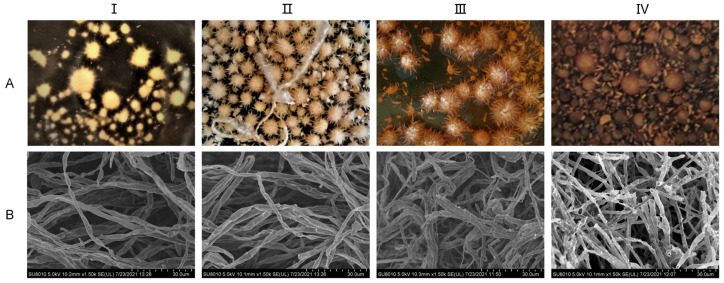
Growth status of *A. ostoyae* mycelia in liquid culture. (**A**): Morphology of the liquid-cultured mycelia. (**B**): Morphology of the mycelia under a scanning electron microscope. (**I**–**IV**) represent days 4, 8, 12, and 24, respectively.

The liquid mycelia of *A. ostoyae* were cultured in the media described in Section 2.2 for a total of 24 days. As shown in Figure 2, the 4-day mycelium was colorless, transparent, thin, and few in number; the 8-day mycelium appeared as a beige spiny ball, the number of mycelia had increased, and the mycelium itself had a certain spiral fold. The 12-day mycelium was a light honey color in which part of the burr had disappeared, the mycelium was loose and appeared bent and entangled when observed under electron microscopy. The 24-day mycelium was a honey color and had a smooth surface, the number of mycelia had decreased, and the mycelium underwent autolysis; the broken mycelia were visible under electron microscopy. That is, after 4–8 days of culture, mycelia began to form, and after 12 days, the mycelia tended to mature. Aging began at 24 days.

### 3.3. UHPLC–QTOF/MS-Based Secondary Metabolite Analysis

**Figure 3:**
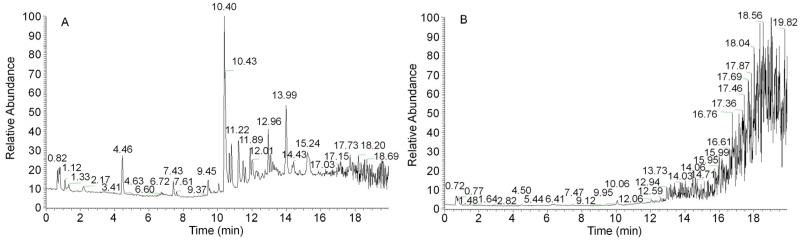
UHPLC–QTOF/MS-based total ion flow diagrams of the *A. ostoyae* mycelium ethanol extract (AOME) in positive ion mode (**A**) and negative ion mode (**B**).

Because the synthesis of secondary metabolites lags behind mycelial growth, the 12-day mycelia were selected for secondary metabolite detection and analysis. A sample from the 12-day mycelia was prepared to obtain an ethanol extract. Figure 3 shows the complete ion chromatograms of the ethanol extract of *A. ostoyae* mycelia (AOME) in both positive and negative ion modes. By mass spectrometry analysis, a total of 1273 compounds were identified by comparison with the database and the literature. These compounds included 18 melleolides, 14 terpenes, 52 flavonoids, 8 coumarins, 4 sphingolipids, 20 alkaloids, 6 lignins, 4 phenols, 173 heterocycles, and 974 other compounds.

### 3.4. Network Pharmacology Analysis

#### 3.4.1. Screening of Active Ingredients

To clarify which substances in the AOME play a key role in inhibiting gastric cancer, network pharmacology analysis was performed. Functional compounds with good oral bioavailability were selected using the SwissADME platform combined with the UHPLC-QTOF/MS data. According to the pharmacokinetic data (high GI absorption value) and drug-likeness index (satisfying more than two conditions), 82 bioactive ingredients in AOME were selected, as shown in Table 1. These active compounds included hydroxylamines, aromatic ethers, flavonoids, terpenes, steroids, coumarins, sesquiterpene lactones, melleolides, alkaloids, and others.

#### 3.4.2. Identification of Potent Substances and Disease Targets

The target information of the bioactive components was screened against the Swiss Target Prediction database. After eliminating duplicate targets, 328 potential target genes were screened with the 82 bioactive components of the AOME. The potential targets were subsequently converted into gene symbols using the UniProt database to utilize them for network construction and additional biological characterization. A total of 1250 targets were obtained by searching for information related to gastric cancer via the GeneCards, DrugBank, DisGeNET, PharmGKB, OMIM, NCBI, and TTD databases and screening, merging, and eliminating any duplicate results.

#### 3.4.3. PPI Network Construction, Merging, and Analysis

**Figure 4:**
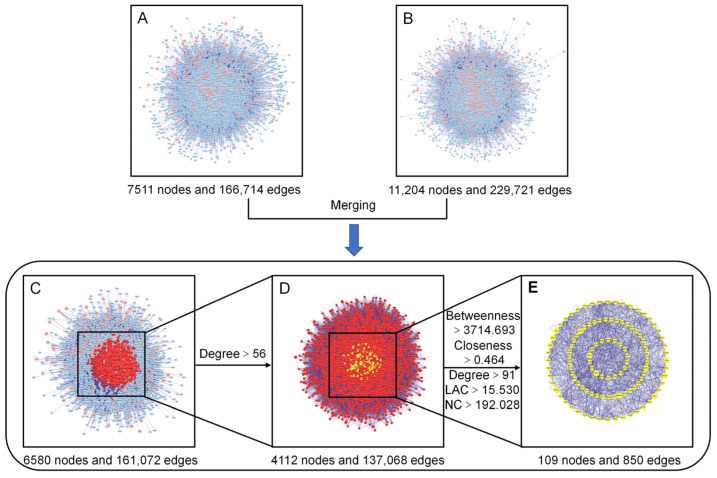
Identification of the core targets of the AOME in gastric cancer. (**A**): Putative target PPI network of the AOME. (**B**): Gastric-cancer-related target PPI network. (**C**): Interactive PPI network of the putative targets of the AOME and gastric-cancer-related targets. (**D**): PPI network of significant proteins extracted from (**C**). (**E**): PPI network of candidate AOME targets in gastric cancer extracted from (**D**).

To gain a more systematic insight into the molecular mechanisms of the diseases and functions in which the specific proteins were involved [27], we constructed an intuitive PPI network of the presumed targets of the AOME (Figure 4A), which contained 7511 nodes and 166,714 edges. The PPI network specifically constructed for gastric-cancer-related targets included 11,204 nodes and 229,721 edges (Figure 4B). The interacting proteins are represented by nodes in the PPI network, while their interactions are represented by edges.

The two aforementioned PPI networks were subsequently merged to discover potential targets of the AOME in gastric cancer, aiding in the elucidation of the possible mechanism of action of the AOME against gastric cancer. The PPI network of the interactions of the AOME with gastric cancer included a total of 6580 nodes and 161,072 edges (Figure 4C). CytoNCA v. 2.1.6 software was subsequently used to analyze the topological properties of the abovementioned merged PPI network. The central network of interactions (Figure 4E) between the AOME and gastric cancer was formed by systematically identifying pivotal genes using five parameters, namely, betweenness, closeness, degree, local average connectivity-based method (LAC), and network centrality (NC) (Figure 4D). The core enrichment modules involved EGFR, IL6, SRC, ESR1, MTOR, MAPK3, PTGS2, MAPK1, and ABCB1.

#### 3.4.4. GO and KEGG Pathway Analyses of the Core Network

**Figure 5:**
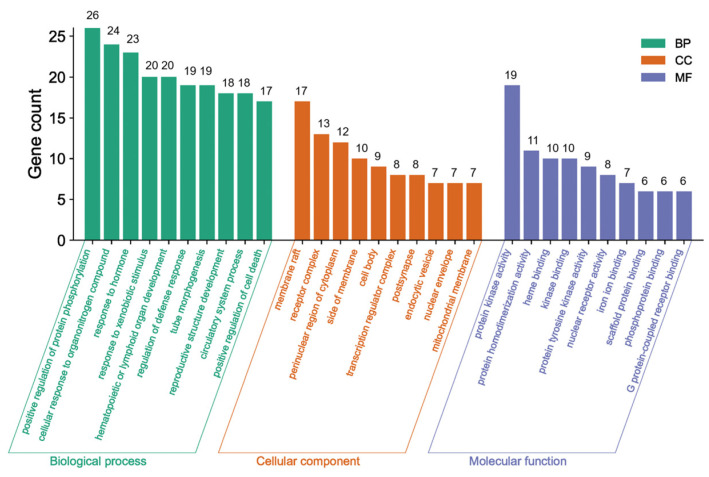
GO enrichment analysis.

The Metascape database was used for further gene classification and enrichment analyses of the 69 common targets. Clusters were created by considering membership, using *p* < 0.01, count number > 3, and enrichment factor > 1.5. The GO enrichment analysis revealed that out of the 1640 GO terms, a total of 69 genes in the core network were significantly enriched. These genes were distributed across different categories, with 1110 in the BP category, 57 in the CC category, and 106 in the MF category. Figure 5 displays the top 10 GO terms.

The green bars in Figure 5 indicate the main BPs, including protein phosphorylation enhancement, cellular response to organonitrogen compounds, response to hormones, response to xenobiotic stimulus, hematopoietic or lymphoid organ development, defense response regulation, tube morphogenesis, reproductive structure development, circulatory system functioning, and cell death promotion. These findings imply established biological impacts on protein phosphorylation, the defense response, and cell death. The most representative CCs represented by the orange bars included membrane raft, receptor complex, perinuclear region of cytoplasm, side of membrane, cell body, endocytic vesicle, transcription regulator complex, post synapse, nuclear envelope, and mitochondrial membrane, which clearly explained the cell composition-related functions. The indigo bars indicate the most important MFs, including protein kinase activity, nuclear receptor activity, heme binding, protein tyrosine kinase activity, scaffold protein binding, phosphoprotein binding, iron ion binding, protein homodimerization, kinase binding, and G protein-coupled receptor binding. These MFs effectively elucidated the molecular-related functions.

**Figure 6:**
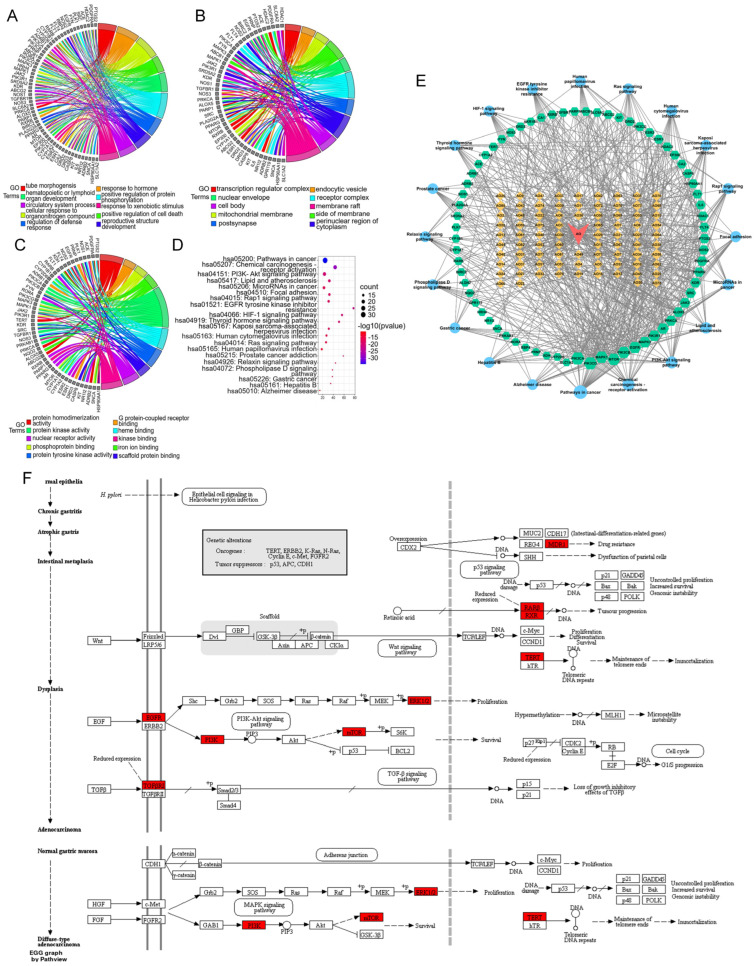
Chord diagram showing the top 10 GO terms in the (**A**) biological process, (**B**) cellular component, and (**C**) molecular function categories. (**D**): Bubble chart of the top 20 KEGG pathways in which the common targets are enriched. (**E**): Drug–compound–target–pathway network. Red inverted triangle (AO), AOME; orange diamonds (AO1–AO82), the potential active ingredients of the AOME listed in Table 1; green hexagons, common targets; blue dots, KEGG pathways. The magnitude of each node represents the magnitude of the d-value of the topology parameter. (**F**): Signaling diagram of the pathways associated with gastric cancer.

A chord diagram of the top 10 enriched genes according to the GO results is shown in Figure 6A–C. In addition, the KEGG analysis revealed a total of 165 pathways (Figure 6D), including cancer, chemical carcinogenesis—receptor activation, the PI3K-AKT signaling pathway, microRNAs in cancer, focal adhesion, the Rap1 signaling pathway, resistance to EGFR tyrosine kinase inhibitor, the HIF-1 signaling pathway, the thyroid hormone signaling pathway, the Ras signaling pathway, the relaxin signaling pathway, the phospholipase D signaling pathway, and gastric cancer.

Based on the findings from the GO and KEGG pathway enrichment, the active components of the AOME may collectively impede the progression and occurrence of gastric cancer by affecting diverse metabolic pathways. This observation aligns with the extract’s distinctive multicomponent and multitarget attributes.

#### 3.4.5. Drug–Compound–Target–Pathway Network Analysis

By merging the results of the GO and KEGG pathway enrichment analyses, a network comprising drugs, compounds, targets, and pathways was constructed that included the previously mentioned bioactive substances, the signaling pathways, and the shared targets (Figure 6E). The network contained 82 active ingredients, 69 action targets, and 20 important signaling pathways. The results suggested that the AOME can inhibit the development and progression of stomach cancer through its effects on different elements, targets, and pathways. According to the structural analysis, it was initially hypothesized that the AOME may be able to treat gastric cancer through different pathways related to cancer, such as the PI3K-AKT and JAK/STAT signaling pathways; receptor activation, which causes chemical carcinogenesis; microRNAs in cancer; focal adhesion; the Rap1 signaling pathway; resistance to EGFR tyrosine kinase inhibitors; the HIF-1 signaling pathway; the thyroid hormone signaling pathway; the Ras signaling pathway; the relaxin signaling pathway; and gastric cancer. This speculation is supported by the significant presence of targets such as EGFR, IL6, SRC, MTOR, ABCB1, PIK3CA, PIK3B, PIK3GD, PIK3R1, MAPK1, MAPK3, RARB, RXRB, TERT, ESR1, PTGS2, and TGFBR1.

The protein targets were mapped to the signaling map on the KEGG website (*URL*: https://www.kegg.jp/; accessed on 1 January 2023), and the specific signaling pathways associated with gastric cancer were ultimately obtained. Here, the red boxes represent the targets that are enriched in this pathway and the potential targets of the AOME, which also have an increased role in gastric carcinogenesis and development (Figure 6F).

A comprehensive analysis of the network pharmacology data revealed that the AOME is rich in significant functional compounds, including isoliquiritigenin, capsaicin, 4-(dimethylamino)-N-[7-(hydroxyamino)-7-oxoheptyl] benzamide, 3-BHA, parthenolide, cortisol, 7-hydroxycoumarin, and (+)-carpaine. The AOME potentially inhibited the proliferation of gastric cancer cells by modulating signaling pathways such as the JAK/STAT and PI3K/AKT pathways. EGFR, IL6, SRC, ESR1, MTOR, MAPK3, PTGS2, MAPK1, and ABCB1 are the core targets of the AOME-related anti-gastric-cancer gene pathway network.

### 3.5. Molecular Docking

To confirm the relevance of the key functional compounds in the AOME in gastric cancer, we performed molecular docking. We employed the top 10 compounds from the target network of the active ingredients in the extract to act as ligands in the process. The top three targets were selected from both the PPI network for common drug disease targets and the primary pathway target network. Schrödinger software and XP&MM-GBSA analysis were used for the molecular docking analysis (Table 2). The XP docking results were obtained using XP Gscore, where results less than −6 are generally considered to indicate a robust and consistent interaction between the ligand and protein. The MM-GBSA analysis results pertain to MM-GBSA dG Bind. A value less than −30 kcal/mol signifies a lower binding free energy and a secure bond between the ligand and protein.

**Figure 7:**
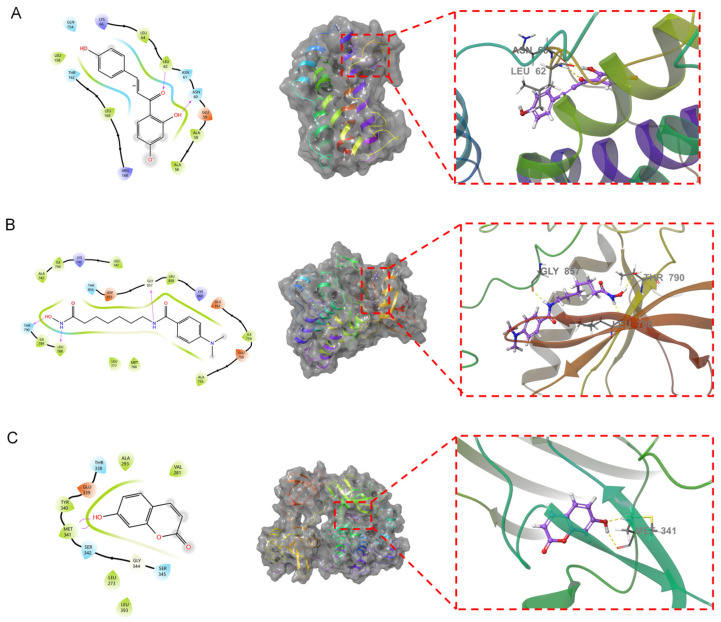
Fusion of the primary compounds and protein receptors. In the figure, the left side shows the 2D molecular docking results with the protein, and the middle shows the 3D results. An enlarged view is shown by the red dotted line on the right. The dotted yellow lines represent hydrogen bonds. The text indicates the residue name. (**A**): Isoliquiritigenin and IL6. (**B**): 4-(Dimethylamino)-N-[7-(hydroxyamino)-7-oxoheptyl] benzamide and EGFR. (**C**): 7-Hydroxycoumarin and SRC.

Isoliquiritigenin demonstrated the best binding with IL6, giving a docking score of −6.426 and an MM-GBSA result of −26.91 kcal/mol. The binding free energy and docking scores were low, indicating that isoliquiritigenin exhibited relatively stable binding with IL6. 4-(Dimethylamino)-N-[7-(hydroxyamino)-7-oxoheptyl] benzamide demonstrated the most superior docking performance with EGFR, with a docking score of −6.092. The MM-GBSA results indicated a value of −43.84 kcal/mol, indicating a low affinity for binding. It can be inferred that EGFR forms a strong stable bond with 4-(dimethylamino)-N-[7-(hydroxyamino)-7-oxoheptyl] benzamide. The interaction between 7-hydroxycoumarin and SRC exhibited the highest docking performance, achieving a docking score of −6.065 and an MM-GBSA result of −31.66 kcal/mol. The binding free energy and docking score were both low, indicating stable binding between 7-hydroxycoumarin and SRC. The therapeutic effect of the AOME on gastric cancer was thus validated via molecular docking. Further analysis was conducted by combining the three most stable combinations, as shown in Figure 7.

Isoliquiritigenin penetrates into the active pocket of IL6, and the IL6 residues LEU158, LEU64, LEU62, ALA58, and ALA56 form hydrophobic interactions with isoliquiritigenin. The residues LEU62 and ASN60 form hydrogen bonds with isoliquiritigenin. 4-(Dimethylamino)-N-[7-(hydroxyamino)-7-oxoheptyl] benzamide effectively penetrates the active pocket of EGFR, forming hydrophobic interactions with residue 3994 and ILE789, LEU788, LEU777, MET766, and LEU858. This ligand also forms hydrogen bonding interactions with residues THR790, LEU788, and GLY857. 7-Hydroxycoumarin binds to the surface of the active pocket of SRC and forms hydrophobic interactions with residues such as MET341, TYR340, ALA293, and VAL281. This ligand forms two hydrogen bonds with MET341.

### 3.6. Cytotoxicity Analysis

#### 3.6.1. Effect on the Proliferative Capacity of MGC-803 Cells

**Figure 8:**
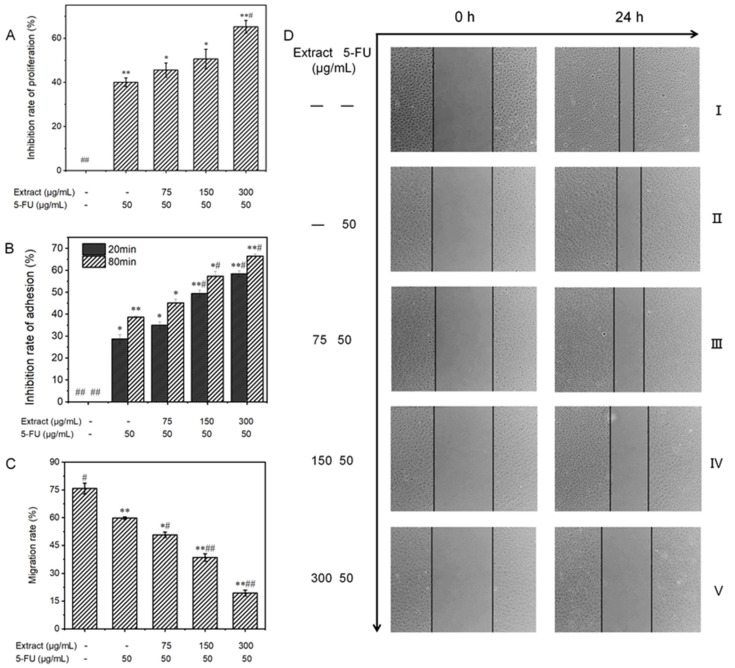
(**A**): The influence of AOME on the growth potential of MGC-803 cells was investigated. (**B**): The effect of AOME on the adhesive capacity of MGC-803 cells was examined. (**C**): The impact of AOME on the migratory capacity of MGC-803 cells was examined. (**D**): Observation of the migration ability of MGC-803 cells (inverted microscope, 40×). Compared to the control group, the experimental group exhibited a notable difference (*p* < 0.05), whereas the model group exhibited an exceedingly significant difference (*p* < 0.001). The same applies below. Group (**I**) was the blank group, Group (**II**) was the positive control group, and Groups (**III**–**V**) represent the combined drug groups with low, medium, and high doses, respectively. */# indicates a significant difference (*p* < 0.05), **/## indicates an extremely significant difference (*p* < 0.001).

To validate the inhibitory effect of liquid-cultured *A. ostoyae* mycelia on gastric cancer cells, 10 key compounds obtained from the web-based pharmacological analysis were compounded according to their concentrations in the extract to prepare the test samples. Since *Armillaria* mycelium extract is a food-grade material, the purpose of this study was to reduce the use of chemical drugs rather than replace them; therefore, the follow-up test mainly involved the combined drug group as the experimental object. The effect of each treatment on the proliferation of MGC-803 cells is depicted in Figure 8A. Compared to the untreated control group, the in vitro proliferation of human gastric cancer cells was distinctly inhibited in the experimental group. This effect increased with the increase in concentration, where the inhibition rate rose from 6.3 ± 1.1% to 28.0 ± 1.4%. The ability of the combination treatment to inhibit the proliferation of MGC-803 cells increased gradually, with the percentage increasing from 45.6 ± 3.2% to 65.2 ± 2.9%. When the concentration of the sample exceeded 150 μg/mL, the cell survival rate decreased significantly compared to the sample group (less than 50%). According to the data presented in Table 3, the low-concentration combined drug group exhibited a CDI of less than 1, while the medium- and high-concentration combined drug groups had CDI values of less than 0.7. These findings indicate that the *A. ostoyae* mycelium ethanol extract (AOME) and 5-FU work in combination to successfully inhibit the proliferation of gastric cancer cells. This synergistic effect was significant as the concentration of the *A. ostoyae* extract increased, and the two components were consumed together. The low- and medium-concentration combined drug groups did not significantly inhibit gastric cancer cell proliferation compared to 5-FU (*p* > 0.05), indicating that the overall effect of the sample combination was weaker (*p* < 0.05). However, the combination of a significant number of different drugs effectively inhibited the proliferation of MGC-803 cells (*p* < 0.05). Since the proliferation of these cancer cells was inhibited, the generation of new cancer cells could be reduced.

#### 3.6.2. Influence on the Adhesion Ability of MGC-803 Cells

The reduced adhesion of cancer cells can effectively prevent cell growth and metastasis. The CCK-8 method, which indirectly reflects the number of living cells via the measurement of light absorption, can be used for cell adhesion analysis. Figure 8B shows that the experimental regimens effectively inhibited the in vitro adhesion of MGC-803 cells in both a dose- and time-dependent manner. In the sample groups, MGC-803 cell expression was inhibited after 20 and 80 min of in vitro culture compared to that in the control group, and the inhibitory effects increased with increasing concentrations. The inhibitory effects in the combination groups also gradually increased, with the inhibitory effects in the low-concentration group increasing from 35.0% to 45.1%, those in the medium-concentration group increasing from 49.4% to 57.3%, and those in the high-concentration group increasing from 58.4% to 66.4%. Therefore, the inhibition of cell adhesion was both concentration- and time-dependent.

#### 3.6.3. Influence on the Migratory Ability of MGC-803 Cells

**Figure 9:**
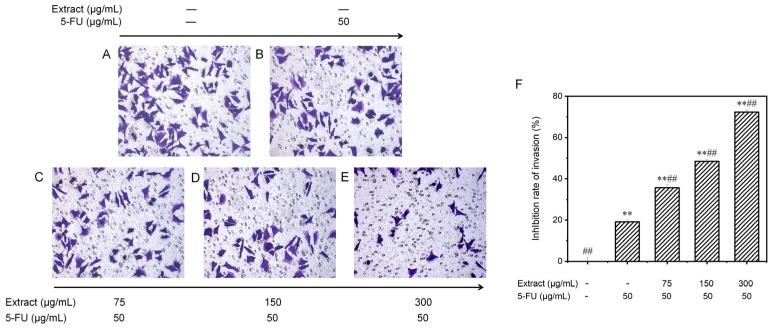
Examining the influence of AOME on the invasion of MGC-803 cells. The invasive potential of MGC-803 cells was assessed using inverted microscopy (100× magnification). The experimental groups were as follows: Group (**A**) received no treatment; Group (**B**) served as the positive control; and Groups (**C**–**E**) were administered different doses of the combined drug (low, medium, and high, respectively). (**F**): Effect of AOME on the invasion ability of MGC-803 gastric cancer cells. **/## indicates an extremely significant difference (*p* < 0.001).

A reduction in the migratory ability of tumor cells may be able to inhibit the infiltration and metastasis of tumors. The scratch method can be used to measure cell motility, and the degree of scratch healing and the migration rate were determined for each group of MGC-803 cells, as shown in Figure 8C and Figure 9. No noticeable alterations in scratch width were observed in any of the groups prior to administration (0 h). Images taken under an inverted microscope after 24 h of incubation are shown in Figure 8D. The cells in the blank control group displayed significant migration toward the original scratch area, producing a narrower scratch with a migration rate of 75.8 ± 3.0%. Additionally, the degree of migration to the scratch area was reduced in the low-concentration combined drug group. Moreover, there was a significant and more obvious decrease in migration to the scratch area in the high-concentration combined drug group. The migration rate decreased from the low-concentration combination drug group (50.8 ± 1.4%) to the medium-concentration combination drug group (38.5 ± 2.0%) and, ultimately, to the high-concentration combination drug group (19.4 ± 1.6%), which showed a significant difference (*p* < 0.001). In the group treated with 5-FU, the rate of cell migration was 59.8 ± 0.7%. Hence, compared to the positive control group, the combined drug groups exhibited significantly more gastric cancer cell migration (*p* < 0.05), with the high-dose combined drug group showing even greater suppression (*p* < 0.001).

#### 3.6.4. Effect on the Invasion Potential of MGC-803 Cells

Invasion precedes tumor metastasis. To observe the invasion ability of MGC-803 cells, the transwell technique was used to assess cell movement traits at low to high nutrient levels. The cells were treated with the blank control, 5-FU (the positive control group), or various concentrations of the combined drugs (Figure 9). The number of invading cells gradually decreased with the increasing drug dose. Figure 9F displays these findings. In the control group without treatment, there were 95.4 ± 3.8 invading cells; in the positive control group, there were 77.2 ± 2.2 invading cells; and in the groups treated with different concentrations of the drug (low, medium, and high), there were 61.4 ± 3.1, 49.2 ± 2.3, and 26.4 ± 2.4 invading cells, respectively. Compared to those in the control group, the invasion capacity of the MGC-803 cells cultured in vitro was partially inhibited in all the groups treated with the combination drugs. Moreover, the inhibitory effect was enhanced with increasing concentrations, as the inhibition rate increased significantly from 35.6 ± 1.4% to 72.3 ± 1.1% (*p* < 0.001). The drug combination exhibited significantly greater effectiveness than 5-FU alone in suppressing the invasion of gastric cancer cells (*p* < 0.001).

#### 3.6.5. Study of the Mechanism of the Extract on MGC-803 Cell Apoptosis

Apoptosis is the process of active cell death that occurs during morphological changes and is an important way for the body to eliminate senescent, damaged, and aberrant cells. Apoptosis is regulated by proto-oncogenes and oncogenes and their products, where the key enzyme caspase-3 induces apoptosis and protein substrate cleavage.

**Figure 10:**
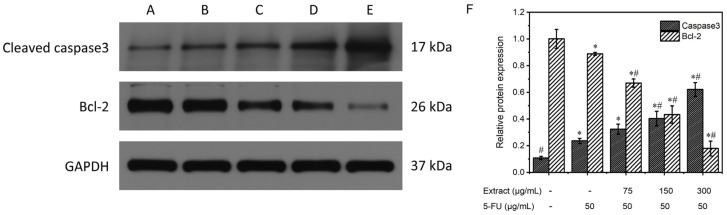
Effects of different concentrations of AOME on protein expression according to the electrophoretic bands from MGC-803 cells. (**F**): Effect of AOME on caspase-3 and Bcl-2 protein expression. The experimental groups were as follows: Group (**A**) received no treatment; Group (**B**) served as the positive control; and Groups (**C**–**E**) were administered different doses of the combined drug (low, medium, and high, respectively). */# indicates a significant difference (*p* < 0.05).

Figure 10 displays the results of the Western blot analysis of the proteins extracted from MGC-803 in every experimental category. The expression of the internal reference, GAPDH, was essentially the same among the five groups. Caspase-3 expression was lower in the control group without treatment but higher in the positive control and the combined drug groups. Furthermore, the combined drug groups exhibited superior clarity compared to the positive control group, and this disparity became more pronounced with increasing concentration. The intensity of the Bcl-2 bands in the untreated control and the positive control groups was greater than that in the drug-treated groups, and the band intensity gradually decreased as the drug concentration increased. The relative protein expression was determined via comparison with the internal reference GAPDH, as shown in Figure 10F. In the blank control group, caspase-3 protein expression was 0.1 ± 0.01, and Bcl-2 protein expression was 1.0 ± 0.07; in the positive control group, caspase-3 protein expression was 0.2 ± 0.02, and Bcl-2 protein expression was 0.9 ± 0.01; and in the groups treated with different concentrations (low, medium, and high) of the combined drug, caspase-3 protein expression was 0.3 ± 0.04, 0.4 ± 0.06, and 0.6 ± 0.05, and Bcl-2 protein expression was 0.7 ± 0.03, 0.4 ± 0.07, and 0.2 ± 0.06, respectively. The combined drug treatment groups demonstrated higher caspase-3 protein expression than the control group without treatment (*p* < 0.05). Furthermore, an increase in the drug concentration correlated with an increased expression of caspase-3. In contrast, compared with that in the blank control group, the expression of Bcl-2 in the combined drug treatment groups was lower (*p* < 0.05). Furthermore, there was an inverse relationship between the expression of Bcl-2 and drug concentration, with higher drug concentrations resulting in lower Bcl-2 expression. In the combined drug treatment group, the expression of the caspase-3 protein was significantly greater than that in the positive control (5-FU) group (*p* < 0.05), whereas the expression of the Bcl-2 protein was lower than that in the positive control (5-FU) group (*p* < 0.05). These findings suggested that the combination treatment had a greater ability to induce apoptosis than the positive control treatment that utilized 5-FU. Reducing the expression of the proto-oncogene Bcl-2 enhances the release of cytochrome C, leading to the activation of caspase-3, a crucial enzyme in apoptosis. These data further demonstrated the proapoptotic effect of the AOME on gastric cancer cells.

## 4. Discussion

*A. ostoyae* was subjected to liquid culture and SEM observations. After 4–8 days of culture, mycelia began to form, and after 12 days, the mycelia tended to mature. Aging began at 24 days. According to Table 1, the characteristic compounds of *A. ostoyae* include hydroxylamines, aromatic ethers, flavonoids, terpenes, steroids, coumarins, sesquiterpene lactones, melleolides, and alkaloids. After a network pharmacology analysis, 10 core functional compounds and nine core targets were found to be involved in gastric cancer. Molecular docking identified three stable complexes.

EGFR, IL6, SRC, ESR1, MTOR, MAPK3, PTGS2, MAPK1, and ABCB1 are crucial components of this network and have been identified as significant targets for treating cancer with the *A. ostoyae* mycelium ethanol extract (AOME). MAPK is a crucial intracellular signaling transmitter that can engage in diverse biological processes, including cellular growth, specialization, and programmed cell death; the immune response; reactions to stress; and defense against infections by phosphorylating nuclear transcription factors and associated enzymes. Compared with that in normal patients, the expression of MAPK family proteins is significantly altered in tumor patients, indicating that MAPK is indeed involved in tumorigenesis and development [28].

Our research revealed that the AOME successfully inhibited the malignant traits of gastric cancer cells by affecting different signaling pathways. The JAK/STAT and PI3K/AKT signaling pathways were confirmed through in vitro studies. The JAK/STAT pathway is associated with cancer and is involved in the growth, development, death, and other crucial biological processes of tumor cells [29,30]. Moreover, the excessive activation of this pathway significantly contributes to the progression of gastritis to gastric cancer [31]. The JAK/STAT signaling pathway can trigger the activation of NF-κB, resulting in the direct or indirect stimulation of malignant tumor behavior [32]. In addition, the expression level of the c-Myc protein is positively correlated with the malignancy, infiltration, and metastatic ability of gastric cancer [33]. JAK/STAT activation can disrupt c-Myc-induced gene expression, leading to the increased expression of related proteins and the impairment of normal cellular metabolism. This ultimately facilitates the progression of gastritis to gastric cancer [34]. The growth of cancer cells is closely linked to the PI3K/AKT signaling pathway [35,36,37]. The interaction between PI3K and EGFR triggers the activation of AKT, which subsequently modulates the function of various downstream targets, including the apoptosis-associated molecules Bax and caspase-3. The cellular processes that are regulated include activities such as cell division, specialization, programmed cell death, movement, and various noticeable traits. In addition, activated AKT can stimulate IKK, leading to the phosphorylation and translocation of NF-κB to the nucleus. The abnormal stimulation of the JAK/STAT signaling pathway results in the activation of the proteins Bcl-2 and c-Myc within the nucleus, while the protein NF-κB is activated via the PI3K/AKT pathway. The cellular experiments demonstrated alterations in key enzymes leading to apoptosis, with a decrease in Bcl-2 expression and an increase in caspase-3 expression. Combined with the network pharmacology results, these findings suggested that the AOME likely inhibits gastric cancer through the JAK/STAT and PI3K/AKT pathways. 

However, the research has some potential limitations. Additionally, there may be specific conditions required for optimal mycelial growth or possible differences in the composition of biologically active compounds in different strains or batches of *A. ostoyae*. In addition, it is uncertain whether the findings can be applied to other strains or types of medicinal mushrooms. Additional research may be required to examine the effects of various strains and types of medicinal mushrooms on different types of cancer. Additionally, the efficacy of the AOME against gastric cancer needs confirmation through meticulous in vivo and in vitro experiments. These studies should concentrate on examining the upstream proteins responsible for activating each pathway, as well as the downstream proteins, including those within the nucleus. Clinical trials are also essential to validate the findings of preclinical studies and to assess the potential benefits and risks in human subjects.

## 5. Conclusions

This paper analyzes the anti-gastric cancer function of AOME using UHPLC-QTOF/MS, network pharmacology, and molecular docking techniques. The results indicate that compounds such as isoliquiritigenin, [(2R,4S,4aR,7aS,7bR),3-formyl-4-hydroxy-6,6,7b-trimethyl-2,4,4a,5,7,7a-hexahydro-1H-cyclobuta[e]inden-2-yl], 3-chloro-6-hydroxy-4-methoxy-2-methylbenzoate, 3-formyl-2a-hydroxy-6,6,7-trimethyl-2,2a,4a,5,6,7,7a,7b-octahydro-1H-cyclobuta[e]inden-2-yl 3-chloro-6-hydroxy-4-methoxy-2-methylbenzoate, capsaicin, 4-(dimethylamino)-N-[7-(hydroxyamino)-7-oxoheptyl] benzamide, 3-BHA, parthenolide, cortisol, and 7-hydroxycoumarin (+)-carpaine can regulate most of the targets and play a significant role in the anti-gastric-cancer effects of the AOME. The JAK/STAT and PI3K/AKT pathways are likely suppressed by the AOME, which leads to inhibitory effects on gastric cancer cells. EGFR, IL6, SRC, ESR1, MTOR, MAPK3, PTGS2, MAPK1, and ABCB1 are the core targets of the AOME in the anti-gastric-cancer gene pathway network. Moreover, the stable binding complexes were identified: isoliquiritigenin to IL6, 4-(dimethylamine)-N-[7-(hydroxyamino)-7-oxoheptyl] benzamide to EGFR and 7-hydroxycoumarin to SRC. This investigation revealed the efficacious components and molecular pathways of the AOME for curing stomach cancer. This study utilized pure maternal bacteria for the artificial liquid culture of the mycelia, an approach that can allow for the large-scale industrial production of mycelia, solving the problem of insufficient natural resources and providing new raw materials for edible mushroom sources, auxiliary functional foods, and drugs against gastric cancer.

## Figures and Tables

**Table 1:** Screened bioactive components of the *A. ostoyae* mycelium ethanol extract (AOME).

Ingredient ID	Ingredient Name	Ingredient ID	Ingredient Name	Ingredient ID	Ingredient Name
AO1	α-Eleostearic acid	AO2	ValylIsoleucine	AO3	Tretinoin
AO4	Suberic acid	AO5	Spinochalcone A	AO6	Saccharin
AO7	Propofol	AO8	Promethazine sulfoxide	AO9	Preclamol
AO10	Penbutolol	AO11	Pazelliptine	AO12	Parthenolide
AO13	Palmatine	AO14	Olodaterol	AO15	N-benzyl-4-(isobutylamino)-2-[4-(tetrahydropyran-3-ylmethyl) piperazin-1-yl] pyrimidine-5-carboxamide
AO16	N-acetyl-2-phenylethylamine	AO17	N-{[(4E)-2-(Hydroxymethyl)-4-(3-isobutyl-5-methylhexylidene)-5-oxotetrahydro-2-furanyl] methyl}-2,2-dimethylpropanamide	AO18	N-[2-(3,4-Dimethoxyphenyl) ethyl]-3-(2-methoxyphenyl)-5-methylpyrazolo[1,5-a] pyrimidin-7-amine
AO19	N-[(9Z)-9-Octadecenoyl] glycine	AO20	N,N-Dimethylarginine	AO21	N,N-Dibutylethanolamine
AO22	Momelotinib	AO23	Metirosine	AO24	Lonchocarpol E
AO25	Linoleic acid	AO26	L-Glutamic acid	AO27	Leucylleucine
AO28	Jasmonic acid	AO29	Isopropyl 4-[3-(3-chloro-4-methoxyphenyl)-1-phenyl-1H-pyrazol-4-yl]-6-methyl-2-oxo-1,2,3,4-tetrahydro-5-pyrimidinecarboxylate	AO30	Isophorone
AO31	Isoliquiritigenin	AO32	hypophyllanthin	AO33	Histamine
AO34	Heteroflavanone B	AO35	goralatide	AO36	Flavone
AO37	Etiproston	AO38	Estriol	AO39	Embelin
AO40	DMH4	AO41	DL-Arginine	AO42	Decanamide
AO43	Cortisol	AO44	Catalposide	AO45	Capsaicin
AO46	Boldione	AO47	Azelaic acid	AO48	AUDA
AO49	Arachidonic acid	AO50	Anacardic acid	AO51	Ambrosic acid
AO52	Ajugarin I	AO53	8-{3-Oxo-2-[(2E)-2-penten-1-yl]-1-cyclopenten-1-yl} octanoic acid	AO54	7-Hydroxycoumarine
AO55	5α-Dihydrotestosterone	AO56	5-[4-(6-Methyl-4-phenylquinazolin-2-yl) piperazin-1-yl]-5-oxopentanoic acid	AO57	5-[(8aS)-2,5,5,8a-tetramethyl-3-oxo-4a,6,7,8-tetrahydro-4H-naphthalen-1-yl]-3-methylpentanoic acid
AO58	5-(3,4-Dimethoxyphenyl)-4-[(2E)-3-(2-furyl)-2-propenoyl]-3-hydroxy-1-[2-(4-morpholinyl) ethyl]-1,5-dihydro-2H-pyrrol-2-one	AO59	4-Phenylbutyric acid	AO60	4-Hydroxy-N’-[(E)-(4-isopropylphenyl) methylene] benzohydrazide
AO61	4-Hydroxyderricin	AO62	4-(Dimethylamino)-N-[7-(hydroxyamino)-7-oxoheptyl] benzamide	AO63	3-Hydroxypregnane-11,20-dione
AO64	3-Formyl-2a-hydroxy-6,6,7b-trimethyl-2,2a,4a,5,6,7,7a,7b-octahydro-1H-cyclobuta[e]inden-2-yl 3-chloro-6-hydroxy-4-methoxy-2-methylbenzoate	AO65	3-BHA	AO66	2-Cyclododecyl-1-(4-morpholinyl) ethanone
AO67	2,2’-(Dodecylimino) diethanol	AO68	2-(4-Morpholinyl)-8-phenyl-4H-chromen-4-one	AO69	1-Amino-3-cyano-4,6-dimethylpyridine-2-one
AO70	13S-hydroxyoctadecadienoic acid	AO71	12-HHTrE	AO72	1-{2-Chloro-4-[(6,7-dimethoxy-4-quinazolinyl) oxy] phenyl}-3-propylurea
AO73	1-[2-(Dimethylamino) ethyl]-3-hydroxy-5-(3-methoxyphenyl)-4-[(5-methyl-1-phenyl-1H-pyrazol-4-yl) carbonyl]-1,5-dihydro-2H-pyrrol-2-one	AO74	1-(3-Ethoxypropyl)-2-imino-N-(5-methyl-1,2-oxazol-3-yl)-5-oxo-1,5-dihydro-2H-dipyrido[1,2-a:2’,3’-d] pyrimidine-3-carboxamide	AO75	1-(1,3-Benzodioxol-5-yl)-2-(1-pyrrolidinyl)-1-butanone
AO76	[(2R,4S,4aR,7aS,7bR)-3-formyl-4-hydroxy-6,6,7b-trimethyl-2,4,4a,5,7,7a-hexahydro-1H-cyclobuta[e]inden-2-yl] 3-chloro-6-hydroxy-4-methoxy-2-methyl-benzoate	AO77	(E)-3-(4-methoxyphenyl)-1-[2,4,6-trimethoxy-3-(3-methylbut-2-enyl) phenyl] prop-2-en-1-one	AO78	(3S)-5-[(1R,2R,8aS)-2-hydroxy-2,5,5,8a-tetramethyl-3,4,4a,6,7,8-hexahydro-1H-naphthalen-1-yl]-3-methylpentanoic acid
AO79	(3beta,5alpha,23beta)-17,23-Epoxy-5,6-dihydro-3-hydroxyveratraman-11-one	AO80	(11Z)-17-chloro-16-[2-(morpholin-4-yl) ethoxy]-2-oxo-2,3,10,13-tetrahydro-1H-8,4-(azeno)-9,14,1,3,6-benzodioxatriazacyclohexadecine-7-carbonitrile	AO81	(+)-Carpaine
AO82	(+)-Aphidicolin				

**Table 2:** The affinity of the main ligand groups for the receptors was determined for 30 samples.

PubChem CID	Compound	IL6		EGFR		SRC	
		XP G	MM-GBSA	XP G	MM-GBSA	XP G	MM-GBSA
638278	Isoliquiritigenin	−6.426	−26.91	−6.151	−13.27	−6.691	−8.76
44557095	[(2R,4S,4aR,7aS,7bR)-3-formyl-4-hydroxy-6,6,7b-trimethyl-2,4,4a,5,7,7a-hexahydro-1H-cyclobuta[e]inden-2-yl] 3-chloro-6-hydroxy-4-methoxy-2-methyl-benzoate	−4.141	−37.16	−3.718	−23.42	−3.663	−29.57
126031	3-Formyl-2a-hydroxy-6,6,7b-trimethyl-2,2a,4a,5,6,7,7a,7b-octahydro-1H-cyclobuta[e]inden-2-yl 3-chloro-6-hydroxy-4-methoxy-2-methylbenzoate	−4.129	−27.21	−5.111	−27.82	−4.176	−22.54
1548943	Capsaicin	−3.933	−41.97	−4.730	−46.93	−4.803	−33.98
3994	4-(Dimethylamino)-N-[7-(hydroxyamino)-7-oxoheptyl] benzamide	−3.912	−32.72	−6.092	−43.84	−5.047	−30.78
8456	3-BHA	−2.703	−27.16	−3.911	−31.26	−4.092	−22.90
7251185	Parthenolide	−2.376	−25.27	−2.421	−10.37	−2.767	−0.63
5754	Cortisol	−2.232	−21.24	−7.043	−15.80	−3.848	−22.66
5281426	7-Hydroxycoumarine	−1.375	−23.64	−5.274	−27.29	−6.065	−31.66
442630	(+)-Carpaine	−1.212	−6.72	−3.072	−29.27	0.886	−27.36

XP G indicates XP Gscore and MM-GBSA indicates MM-GBSA dG Bind, with units of kcal/mol.

**Table 3:** The affinities of the main ligands for the receptors were determined for 30 samples.

Group	CDI			Mean	Standard Deviation
	1	2	3		
Combination drug low concentration	0.809	0.832	0.827	0.823	0.012
Combination drug medium concentration	0.299	0.341	0.364	0.335	0.033
Combination drug high concentration	0.249	0.301	0.293	0.281	0.028

## Data Availability

Data are contained within the article.

## Abbreviations

*A. ostoyae*, *Armillaria ostoyae*; AO, *Armillaria ostoyae*; AOME, *Armillaria ostoyae* mycelium ethanol extract; BP, biological process; CC, cellular component; CDI, coefficient of drug interaction; ESI, electrospray ionization; 5-FU, 5-fluorouracil; GAPDH, glyceraldehyde-3-phosphate dehydrogenase; KEGG, Kyoto Encyclopedia of Genes and Genomes; MF, molecular function; OD, optical density; UHPLC–QTOF/MS, ultra-performance liquid chromatography quadrupole time-of-flight mass spectrometry; PPIs, protein–protein interactions; CO, cortex; OM, outer medulla; IM, inner medulla; CC, central cavity.

## Appendix A. PCR Amplification Sequences

>M2.ITS4

TGGYGGGTRTCTACCTGATTTGAGGTCAATAGTCAATAAATTGTCCAAGTCAATGGACGGTTAGAAGCTGAATCCTTCTACAAAGTCAAGGGGAAAAATCAAGGAAAGCTAAGCTCGCGCTAAGCAACTCCTCTCTTAGAATCGGACAAGTATCTCCGCAAAGGGAGAAAGCAGAAGGAGCCGTTAAGCACCTTCCAAACCGATCCTAAGCCAGACTCGAAAGCCTGATTAGTTACCACTGTATGATTCGTATTCCGACCCAACTACCAAGGCGTAGATATTATCACAGCCTAGCAGCCAAAGTCAAACGGTTTCTGCTAATGCATTTCAGAGGAGCTGATCTCGTTAGAAACCAGCAAACCCCCATATCCAATCCGCCGCACTCCTAATGAAAGAAGGGGAGATTGAGAATTTAATGACACTCAAACAGGCATGCCCTTCGGAATACCAAAGGGCGCAAGGTGCGTTCAAAGACTCGATGATTCACTGAATTCTGCAATTCACATTAGTTATCGCATTTCGCTGCGTTCTTCATCGATGCGAGAGCCAAGAGATCCGTTGTTGAAAGTTGTATAAGATTTAAAGGACTTGCGTCCCATAAACAAGACATTCTAGACATACAAGAGATTATATAGACATAGACTTGATAGACAAAGGGAGCTCGAAAGCAACCCTTCTAACCGCTCGAGAGCGAAAGTTAATCAAGTCTACAAAGGTGCACAGGTGGATGAATAGAACGCAACACGTCGAACGTGCACATACCCTTTAACAGGTCAGCAACAGTTCTCAATGCTACGATTCAAGTTTCAATAATGATCCTTCCGCAGGTTCACCTACGGAAACCTTGTTACGCTTTTTTTYMYTCYACA

>M2.ITS5

CCTTTGGGACTGCGGAGGWCATTATTGAACTTGAATCGTAGCATTGAGAACTGTTGCTGACCTGTTAAAGGGTATGTGCACGTTCGACGTGTTGCGTTCTATTCATCCACCTGTGCACCTTTGTAGACTTGATTAACTTTCGCTCTCGAGCGGTTAGAAGGGTTGCTTTCGAGCTCCCTTTGTCTATCAAGTCTATGTCTATATAATCTCTTGTATGTCTAGAATGTCTTGTTTATGGGACGCAAGTCCTTTAAATCTTATACAACTTTCAACAACGGATCTCTTGGCTCTCGCATCGATGAAGAACGCAGCGAAATGCGATAACTAATGTGAATTGCAGAATTCAGTGAATCATCGAGTCTTTGAACGCACCTTGCGCCCTTTGGTATTCCGAAGGGCATGCCTGTTTGAGTGTCATTAAATTCTCAATCTCCCCTTCTTTCATTAGGAGTGCGGCGGATTGGATATGGGGGTTTGCTGGTTTCTAACGAGATCAGCTCCTCTGAAATGCATTAGCAGAAACCGTTTGACTTTGGCTGCTAGGCTGTGATAATATCTACGCCTTGGTAGTTGGGTCGGAATACGAATCATACAGTGGTAACTAATCAGGCTTTCGAGTCTGGCTTAGGATCGGTTTGGAAGGTGCTTAACGGCTCCTTCTGCTTTCTCCCTTTGCGGAGATACTTGTCCGATTCTAAGAGAGGAGTTGCTTAGCGCGAGCTTAGCTTTCCTTGATTTTTCCCCTTGACTTTGTAGAAGGATTCAGCTTCTAACCGTCCATTGACTTGGACAATTTATTGACTATTTGACCTCAAATCAGGTAGGACTACCCGCTGAACTTAAGCATATCAWAAAARGGGRRRRAA

>M2.EF595F

GCGWAATCAGCTGATTGTGCCATTCTCATCATCGCTGGTGGAACTGGTGAATTTGAAGCCGGTATTTCCAAGGACGGYCAGACCCGAGAGCACGCCCTCCTTGCCTTCACCCTCGGTGTCAGGCAGCTCATCGTCGCCGTCAACAAGATGGACACCACCAAGGTACGAGATCTATCGTTTTACCTTTTTCCTTAGGCAAATCTGACTGTTATCTCAGTGGAGCGAGGACCGGTTCAACGAAATCGTCAAGGAAACTTCCACCTTCATCAAGAAGGTCGGCTACAACCCCAAGGCCGTCGCTTTCGTCCCCATCTCTGGATGGCACGGTGATAACATGTTGGAGGAGTCCGCCAAGTAAGTCCTTACCTAAGTATGACCAGTGCTGCCTCTTAACGTTCTCTGTAGCATGCCATGGTACAAGGGCTGGACCAAAGAGACCAAGGCCGGTGTCGTCAAGGGCAAGACTCTCCTCGATGCCATTGACGCCATTGAGCCCCCTGTCCGTCCCTCCGACAAGCCTCTCCGTCTCCCTCTCCAGGACGTCACAAAGATCGGA

>M2.EF1160R

TCCGCGAAAGCTGTCGGAGGGAGGACAGGGGGCTCAATGGCGTCAATGGCATCGAGGAGAGTCTTGCCCTTGACGACACCGGCCTTGGTCTCTTTGGTCCAGCCCTTGTACCATGGCATGCTACAGAGAACGTTAAGAGGCAGCACTGGTCATACTTAGGTAAGGACTTACTTGGCGGACTCCTCCAACATGTTATCACCGTGCCATCCAGAGATGGGGACGAAAGCGACGGCCTTGGGGTTGTAGCCGACCTTCTTGATGAAGGTGGAAGTTTCCTTGACGATTTCGTTGAACCGGTCCTCGCTCCACTGAGATAACAGTCAGATTTGCCTAAGGAAAAAGGTAAAACGATAGATCTCGTACCTTGGTGGTGTCCATCTTGTTGACGGCGACGATGAGCTGCCTGACACCGAGGGTGAAGGCAAGGAGGGCGTGCTCTCGGGTCTGGCCGTCCTTGGAAATACCGGCTTCAAATTCACCAGTTCCACCAGCGATGATGAGAATGGCACAATCAGCCTGGGAGGTACCAGTGATCATGTTCTTGTGAAAGTCACGA

>M2.TUBF

AGCTTTCTTCTTMTCTTCTCTGTTTTATGGAGCTGTTCTTGTATTCGCCTCTTTTCAATGCCCCTTTCTTTCTTTTTACCCCTGKCATCTGCTCACATTTGATTTTTTGCGTCTAGACTATAMCGAGGGTGCTGAACTCGTCGACTCTGTTCTGGATGTCGTGCGCAAGGAGGCAGAGGGAACCGACTGCCTGCAAGGTCAGCATAGCAACAAACACTTGTTCTCCTTCTCATGTCGTTATAGGCTTCCAAATCACCCATTCCCTTGGTGGTGGAACCGGTGCTGGTATGGGTACTCTCTTGATCTCAAAGATCAGGGAAGAGTACCCAGACAGAATGATGTGCACGTACTCTGTCGTGCCCAGTCCTAAAGTCTCGGACACTGTCGTCGAGGTACGCTATTTTAAATCGCATTGTATACGTAATGACACCCGAGCCCTAGCCCTACAATGCCACTCTCTCAGTGCATCAACTCGTTGAGAACTCTGATGAAACGTTCTGTATTGATAACGAAGCTTTGTATGATATCTGCTTCCGAACGCTCAAGCTCTCTACACCCACCTACGGCGATTTGAACCACCTGGTCTCCATCGTCATGTCCGGCATAACTACCTGTCTGCGATTCCCCGGTCAGCTAAACTCTGATTTGAGGAAGTTGGCTGTGAACATGGGTGCGGCTCATTTCAATTGTGATTTGCTCACGCTGACCGAGTGTATTCTAATTAGTTCCTTTCCCCCGTCTGCATTTCTTCATGACCGGTTTTGCACCTTTGACTGCTCGCGGAAGCCAGCAATACCGTGCCGTGACAGTTCCCGAACTCACGCAACAGATGTTCGATGCAAAGAACATGTGGGCTGCCTCA

>M2.TUBR

GCGGCMCTGATTGCGTGAGTTCGGGACTGTCACGGCACGGTATTGCTGGCTTCCGCGAGCAGTCAAAGGTGCAAAACCGGTCATGAAGAAATGCAGACGGGGGAAAGGAACTAATTAGAATACACTCGGTCAGCGTGAGCAAATCACAATTGAAATGAGCCGCACCCATGTTCACAGCCAACTTCCTCAAATCAGAGTTTAGCTGACCGGGGAATCGCAGACAGGTAGTTATGCCGGACATGACGATGGAGACCAGGTGGTTCAAATCGCCGTAGGTGGGTGTAGAGAGCTTGAGCGTTCGGAAGCAGATATCATACAAAGCTTCGTTATCAATACAGAACGTTTCATCAGAGTTCTCAACGAGTTGATGCACTGAGAGAGTGGCATTGTAGGGCTAGGGCTCGGGTGTCATTACGTATACAATGCGATTTAAAATAGCGTACCTCGACGACAGTGTCCGAGACTTTAGGACTGGGCACGACAGAGTACGTGCACATCATTCTGTCTGGGTACTCTTCCCTGATCTTTGAGATCAAGAGAGTACCCATACCAGCACCGGTTCCACCACCAAGGGAATGGGTGATTTGGAAGCCTATAACGACATGAGAAGGAGAACAAGTGTTTGTTGCTATGCTGACCTTGCAGGCAGTCGGTTCCCTCTGCCTCCTTGCGCACGACATCCAGAACAGAGTCGACGAGTTCAGCACCCTCGGTATAGTCTAGACGCAAAAAATCAAATGTGAGCAGATGACAGGGGAAAAAAGAAAGAAAGGGGCATTGAAAAGAGGCGAATACAAGAACAGCTCCATAAAACAGAGGAAGAGTAAACAGAGACTCACGCCCTTTCGCCCAGTTACCCGCACCA
